# Proceedings of NADEP Diabetes Foot Conference held at Lahore, Pakistan (August 10-12, 2018)

**DOI:** 10.12669/pjms.345.16509

**Published:** 2018

**Authors:** Shaukat Ali Jawaid

LAHORE: National Association of Diabetes Educators of Pakistan in collaboration of Baqai Medical University, Baqai Institute of Diabetes and Endocrinology and Pakistan Working Group on Diabetic Foot organized its conference at Lahore from August 10-12, 2018. It was very well attended by Diabetologists, endocrinologists and family physicians interested in diabetic foot care. Saving Feet by implementation of guidelines in clinical practice was the theme of the conference. **Prof. Khalid Masood Gondal** Vice Chancellor of King Edward Medical University was the chief guest in the inaugural session. Speaking on this occasion he said that no surgeon is ever interested in doing amputation surgery for diabetic foot. Early diagnosis of diabetic foot, timely management including debridement and patient education is extremely important. College of Physicians & Surgeons Pakistan, he said, has now started postgraduate training programmes of Fellowship in Endocrinology. He suggested that each medical college and medical university should establish professorial unit of endocrinology. Proper diabetic foot care will save lot of amputations. Diabetes he further stated affects all organs of the body; hence we need to intensify our efforts for prevention of diabetes.

**Prof. A. Basit** Director of BIDE speaking on the occasion said that after the establishment of Diabetic Foot Care clinic, they have been able to reduce the amputation rate at BIDE by 75%. Since 2007 they have established one hundred fifteen diabetic foot clinics and it has helped reduce amputation rate by 50%. We are aiming at establishing three thousand diabetic foot clinics all over the country at primary care centers which can then refer the patients to tertiary care centers. At present there are about twenty-five lac diabetic foot patients and with the 10% amputation rate, we are having two and a half lac amputations every year. Mortality after diabetic foot amputation is very high almost 70%. NADEP is busy in promoting diabetes education and now Ministry of Health has also accepted to recognize the role of Allied Health Care Professionals like Diabetes Educators and Podiatrists. Their degrees should be recognized. We need about three hundred secondary care diabetes centers in Pakistan. Treatment of diabetic foot ulcer is very expensive and according to a recent survey almost 25% of our population is either diabetic or in pre-diabetic stage.

**Figure:**
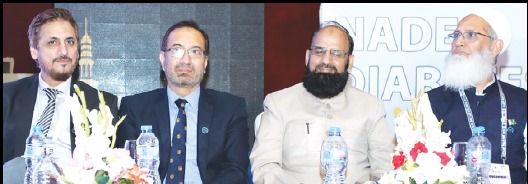
*Prof. Khalid Masood Gondal Vice Chancellor of KEMU was chief guest at the inaugural session of NADEP Foot Congress 2018. Picture shows him sitting on the dais alongwith Dr. Zahid Miyan, Prof. Bilal Bin Younis and Prof. Abdul Basit*.

**Dr. Zahid Miyan** in his address said that NADEP was a unique platform where people with diabetes work together. It has nurses, doctors, podiatrists, patients with the main aim of improving the diabetes care. Its objectives are standardization of diabetes care, education and management. We are interested in mass education and have prepared national guidelines. We will share our knowledge in diabetic foot care management thereby saving feet. We are developing practical skills thereby enhancing national coherence.

Earlier Prof. **Bilal Bin Younis** in his welcome address highlighted the challenges being faced by Pakistan in diabetes in general and diabetic foot care in particular. According to latest survey by BIDE almost every fourth Pakistani is suffering from diabetes. Management of diabetic foot is a very complex one. We have to prevent diabetes and tackle this challenge, he added.

**Footwear and Offloading**

The first scientific session on August 11th was chaired by Dr. G.Abbas Zulfikar from Tanzania along with Prof.Salma Tanveer while Dr.Fauzia Moyeen moderated this session. **Prof. A. Basit** Director BIDE was the first speaker who talked about where are we now as regards implementation of Diabetic Foot Care Guidelines. These guidelines, he said, were for the first time prepared in 2007. The International Working Group on Diabetic Foot which had prepared these guidelines has very few representatives from Asia and Africa but mostly from the West who know little as to what is happening in these parts of the world. According to reports almost one million people lose their legs annually and one leg is lost almost every twenty second. Till 1996, there was no Diabetic Foot Clinic in Paksitan. We have about four million people with diabetic foot ulcers. The national diabetes survey which we have conducted recently shows that there are 26.1% people with diabetes and 14.4% of people are in pre-diabetes stage. There are about twenty seven lac people with diabetic foot.

**We are planning to have Diabetes Registry for Type-1, Diabetic Foot Registry & Gestational Diabetes Registry-Prof. Basit**

We have started training diabetic foot care assistants and diabetes educators which has enabled us to provide cost effective diabetes healthcare. When ulcer develops, the cost of care goes up manifold. So far, Prof. Basit said, we have trained 126 diabetic foot care assistance. They work under supervision of primary care physicians. We have documented all what we do. We have prepared guidelines for guidance. It is important to develop local guidelines and they should be used. World literature also shows that not more than 30% of people with diabetes have HbA1c of below 7. We have developed guidelines for self blood glucose monitoring (SBGM). We have developed Guidelines for Injection Techniques for insulin. These are evidence based guidelines and now IDF has also recognized it. So far we have published seventeen papers on diabetic foot. We have developed economical off loading devices for patients with diabetic foot. We have developed two, five and ten year’s plans. We meet on every Wednesday to discuss diabetic foot cases from different centers. Some diabetic foot clinics have started producing footwear. In five years time we hope to establish six hundred diabetic foot clinics and in ten years time their number will increase to three thousand all over Paksitan. We have now started online Certificate Course. In future we will share data from different countries. At present we are faced with lot of problems and challenges while doing survey. On every Wednesday at 9.00 AM different centers can share their patients with us and we can discuss their management. We hope to start diabetic foot e clinic and all this is directed at reducing the amputation rate. In this group about twelve diabetes tertiary care centers work together under the auspicious of Advisory Board for Care in Diabetes (ABCD). It is important to prevent ulcer in the first place so that there are no amputations. Footwear production has started in five cities. We are planning to have a Diabetes Registry for Type-1, Diabetic Foot registry, Gestational Diabetes Registry, Prof. Basit added.

**Dr. Gulapar Srisawasdi** from Thailand was the second speaker in this session who talked about Footwear and Offloading to prevent and heal foot ulcers. She pointed out that we need protection foot wear and off loading devices because of peripheral neuropathy, vascular occlusion, dry deformed feet, infections, amputations. Many patients with diabetes in Thailand, she said, have inappropriate foot wear and are in the habit of walking barefoot at home. People mostly war slippers or sandals which is a major cause of foot ulceration. She also talked about total control orthosis, decreased shearing force and limited motion of affected joint, weight distribution, shock absorption and protecting the pathologic area in the feet. Best results are achieved with proper shoes.

**Footwear for offloading is well accepted concept to promote ulcer healing and prevent recurrent ulcer-Dr. Gulapar**

Speaking about the limit of motion and functions, she said, rocker bottom soles improve stability. They relieve pressure to specific area, these shoes can be modified and patients can also use their own shoes. These off loading devices relieve pressure to specific area, replace motion and improve gait, decrease shock. We can prepare modified proper shoes for people with diabetes. These shoes need to fit the life style, local culture and be financially affordable. Speaking about D-Foot International she said its goals include providing standardized low cost, durable and acceptable footwear to all people with diabetes and prevention of foot ulcers and amputations utilizing footwear as the major tool. Other factors which can cause ulcer include moisture, swollen foot and friction. Shock proof material is used in the preparation of these shoes. We need therapeutic footwear for people with PAD (Peripheral Arterial Disease) and foot deformity. These shoes should also fit people with peripheral neuropathy. 2015 IWGDF Guidelines recommend that in case of no neuropathy, one can use off-the shelf footwear but for peripheral neuropathy it is essential to take extra care when selecting or being fitted with foot wear. However, therapeutic foot wear is needed for peripheral neuropathy with PAD and peripheral neuropathy and history of foot ulcer or LE amputation. These guidelines also calls for instructing at risk patient with diabetes to wear properly fitting footwear to prevent a foot ulcer. One should not prescribe and instruct a patient with diabetes not to use conventional or standard therapeutic shoes to heal a plantar foot ulcer. Talking about offloading footwear for healing she mentioned about total contact cast, Ankle foot orthosis, Foot orthosis, shoe modification and custom molded shoes. She then shared some case histories of patients with diabetic foot. Given holistic care, they had good diabetes control. They were imparted self care education. We need good Diabetic Foot Care team which will avoid amputation in 85% of patients. She also showed prefabricated Knee-high device with inappropriate foot-device interface. We have different needs and different goals hence we need better communication and multidisciplinary team to manage diabetic foot. We need to talk together in the same language. Our hospital in Thailand, she said, receives about ten thousand new cases of diabetic foot every year. We started Diabetic Foot care service twenty years ago with just two people. Now we offer Bachelor and Master’s degrees. We have many international students and those interested can contact our international department at the hospital. She concluded her presentation by stating that footwear for offloading is a well accepted concept to promote ulcer healing and to prevent recurrent ulcer. Foot wears management with proper fitting play an important role in off loading. Proper wound care along with proper footwear is the keys to heal and prevent ulcer and multidisciplinary approach was the most important aspect. Dr. Zulfikar Abbas and Prof. Basit commended the work being done by Dr. Gulapar and her team which was a great achievement.

## Workshops

This was followed by different workshops on early detection of feet at risk, putting the feet in right shoes, foot infection-identification and assessment besides vascular assessment and diagnosis. The facilitators included **Dr.Asmat Nawaz, Prof.Salma Tanveer and Dr.Riaz Memon, Dr. Saiful Haque, Awn Bin Zafar**. During the workshops it was pointed out that outcome is different in different grades of diabetic foot infections. It is important to identify infections, then treat and manage it. At times ulcers heal and go in remission and it needs continuous care. For vascular assessment history and clinical examination is important. Routine investigations are not reliable in many cases and they need to be supplemented by bedside tests alike Ankle Brachial Index, bedside vascular test, toe brachial test. These tests do have their own limitations. Non healing ulcers then progress to gangrene and eventual amputation.

**Next Generation Therapeutic Options**

**Dr. Atif Munir** from Fatima Memorial Hospital Lahore was the guest speaker at the corporate symposia on next generation therapeutic options wherein he highlighted the safety and efficacy of Ryzodeg- new insulin on the horizon. He opined that for optimal insulin regimen, it has to provide maximum efficacy, should need minimum number of injections, it should be a more user friendly device with minimum hypoglycaemia episodes. He then discussed in detail the clinical evidence of efficacy of Ryzodeg which is a 70/30 combination of two analogues insulin Degludec/InsuliAspart. Its half life is about twenty five hours. A study done in Type-2 diabetes showed that it was in no way inferior to existing insulins, offer safety and efficacy. Moreover HbA1C reduction is much better with this insulin and much less dose is required. It also offers the advantage that one can use it on any patient or switch over any patient to this insulin any time and this switch over is very easy. **Dr. Uzma** pointed out that she has used it and while switching over it is advisable to use lower dose. One should start it with the largest meal in the day to begin with. Its cost is two times as compared to other insulins and this has to be taken into account while prescribing this insulin. However, one should also remember that cost of treating complications is much higher, she remarked. Speaking about the safety of this analogue Dr.Atif Munir said that one has to take the same precautions which are needed with all other analogues. It provides significantly better FPG control, offers significantly better SMPG control before and after breakfast, significant reduction in hypoglycaemia. Responding to questions from the audience he said that there have been no studies in CKD patients and there are no differences in renal disease and chronic liver disease. **Dr. Imtiaz Hassan** who was chairing the session remarked that it was a new wonderful product. Half life of basal insulin, he remarked is dose dependent.

**Therapeutic foot wear is needed for peripheral neuropathy with PAD and peripheral neuropathy and history of foot ulcer or LE amputation**

**Dr. Kristein Van Acker** from Belgium spoke on Time to Implement Guidelines- a Global Perspective. Making a presentation through video link she opined that it is upto the healthcare professionals to decide when where and how to implement these guidelines. Guidelines are cost effective, are like a map where to go and implementation is the journey… IWGDF has divided the world in seven regions spread over one hundred fifty countries with one hundred eighty representatives.

She commended BIDE for starting the Diabetic Foot Care Assistants programme. We have so far trained four thousand healthcare professionals in seventeen countries in diabetic foot care. We are trying to bridge the gap between available and affordable diabetic foot care. She also referred to the podiatry assistant’s programme, D. Foot International, Footwear programme, basic surgery programme, foot tracker care, patient awareness and politician’s awareness programme. Our aim is to have footwear for every diabetic foot. Almost 80% of people with diabetes, she said, live in low and middle income countries and it is essential that they take care of their diabetes. She also talked about the fast track pathway for diabetic foot ulceration and commended the diabetic foot care services made available in Pakistan by BIDE which was very inspiring. She emphasized the importance of first line foot care, limb saving surgery, adequate foot care and podiatry care. Education of politicians, she emphasized, was as important as of patients with diabetes and healthcare providers.

**Prof.Basit** remarked that Diabetic Foot Care Assistants needs to be recognized by the regulatory bodies. We plan to establish Diabetic Foot e Clinic where people can share their diabetic foot cases. We have proposed that let the Ministry of Health run this programme from Islamabad and we would provide the experts. Masters programme in podiatry needs to be started. He suggested that we can link together with Dr.Gulapar in Thailand and work together.

**Foot wears for every diabetic**

This was followed by a presentation by **Dr.Zahid Miyan** on Foot wear for every diabetic. He pointed out that every twenty second, a limb is lost. Patients should present with neuropathy before ulcer develops as there is a window of prevention. This is the time to prevent amputation. He then talked about the history of ulcer, diabetic foot ulcer, off loading plantar pressure, locally made off loading devices. Shoes can help in protection and sandals are most cost effective. Foot ulcers can be prevented. Studies have shown that 80% of patient does not inspect their feet daily and 70% have no proper nail cutting and 90% do not use proper foot wear. Now we have established risk assessment clinic. Mr. Nelson from Netherlands conducted the train the trainers programme. We have started foot wear manufacturing facility providing low cost standardized durable footwear. These shoes are available at cost of Rs. 1400-3000. Cost at every center will be the same for these durable acceptable shoes. During the last one year we have done risk assessment of 29,207 patients at ten centers including BIDE. Almost 3,459 foot wear facilities were availed and 980 patients wear these shoes for about 50% of the time. Among the low risk one hundred thirty patients had no ulcer, in the moderate risk 241 patients had no ulcer and in high risk 537 patients had no ulcer. Post ulcer group included seventy two patients of which three were in very high-risk, nineteen had recurrent ulcer and 53 had no ulcer.

**During last one year 29,207 patients were screened at risk assessment centers, 3459 patients availed the footwear facility-Dr.Zahid Miyan**

Speaking about the problems and their possible solutions Dr.Zahid Miyan said that to overcome lack of awareness a structured education programme for patient, family members needs to be started. Corporate partnerships are needed to finance and offer donations for making the footwear affordable. These shoes should have attractive designs, offer different colour choices and to avoid inconvenience, these purpose built shoes should be provided after trial on the same day. We are also faced with Lack of space, Lack of staff and official approval. Footwear technicians are contractual employees and it is difficult to retain them. We need full time dedicated teams and in the initial stages it is extremely difficult to meet operating expenditures. Footwear for every diabetic, Dr.Zahid Miyan said is a new concept in this part of the world. With the collaboration of other centers, we have been able to initiate this concept. Further expansion of footwear facility network across the country is required. We need to focus on prevention of foot ulcer and allocation of resources for this. Therapeutic shoes will be coming soon and we also plan to offer Sandal for Hajj/Umra which will be manufactured soon besides running a footwear assistant course. As regards new development, this programme will be replicated in other low resource regions. Pakistan and Bangkok is the chair of this group. New partnership members are needed for this project.

Management strategies include metabolic control, antibiotics, podiatry, off loading devices and surgical management. Ten new risk assessment clinics will be established. We will promote the low cost and durable footwear manufacturing technique. Ten foot care assistants and ten cobblers from BIDE will be trained besides training one foot care assistant and one cobbler from each project center will also be trained.

**Figure:**
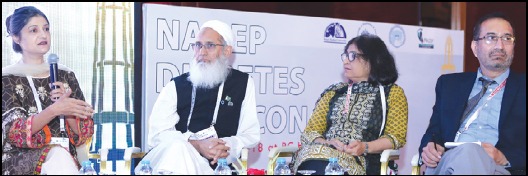
*Dr. Uzma Khan, Prof. Abdul Basit, Prof. Ambar Malik, Prof. Bilal Bin Younis chairing one of the sessions during the NADEP Foot Congress 2018 held at Lahore*.

**Dr. Tarek Fiad** from UAE talked about “Can innovations ease transition to injectable therapies”. An ideal injectable therapy, he said, should have an overall lower rate of hypoglycaemia, offer meaningful decrease in HbA1C levels, lead to weight loss and offer cardiovascular safety. He then discussed the cardiovascular safety of Dulaglutide and the cardiovascular benefits of GLP-1 in detail. It is restricted to those with established cardiovascular disease. It requires once weekly dose, no dial to dose, and no missing and hidden needle. The needles are disposable. When compared with Liraglutide, both were found to be effective. In Type 2 diabetics it results in no weight gain, they have less hypogylcaemic episodes. Weight reduction is better with Dulaglutide; it has no significant cardiovascular adverse events. Replying to questions he said that it has not been tested in children, gestational diabetes, hence it is not recommended.

**Charcot Arthropathy**

**Prof. Bilal Bin Younis** discussed Charcot Arthropathy. He pointed out that once amputation takes place it has a 30% mortality which increases to 50% after three years and 70% after five years. He emphasized about comprehensive history and risk factors to screen these patients. It is physician’s responsibility to inspect the foot, ensure good blood sugar control and educate the patient. According to Prof.Carl Baker only 14% doctors ask the patient to take off the shoes and socks to inspect the foot. Bulge in the center shows Charcot foot and diabetes is a major risk factor for Charcot Arthropathy. One should pick up these cases early to save foot when there is mild swelling and no deformity. Moderate swelling is followed by extreme swelling and more temperature which are signs of effusion. Do laboratory and radiological investigators. A good radiologist can show it. Flat foot is a Charcot foot while the feet with some angle are normal feet. In acute phase the patient should be told not to bear weight. In stage two and three the patient should be advised bed rest. Monitor the patient and opt for non operative casting. Monitor for three to six months and the patient should be advised to change the shoes after every six to twenty four months. It may take one to two years to heal completely. Bring the HbA1c to less than seven. Charcot Arthropathy is a progressive but not an uncommon problem and it has a potential risk hence efforts should be directed at preventing disability and amputation. He also talked about recurrent ulcers. Total destruction of a weight bearing joint is marked by bony destruction, bone resorption and eventual deformity. Treatment consists of casting, shoes, bracing and orthotics besides surgical procedures. There is a need to have a special care of persons with diabetes. Early identification of risk factors may prevent occurrence of Charcot deformity and the patient must be educated about foot care. This condition is most often overlooked though it is a serious limb-threatening complication of diabetes. Deformity is associated with duration of diabetes. It has potential risk of permanent disability, amputation and recurrent ulcers. Neuropathy, HbA1c and duration of diabetes are the predisposing risk factors for Charcot osteoarthropathy, he added.

During the discussion **Dr. Zulfikar Abbass** pointed out that treatment of ulcers with maggots is still under experiments like many other things but we have to keep the treatment simple and effective. **Dr. Uzma** remarked that one can have cultures for different reasons. We must use valid therapy and everything cannot be tried on every patient.

**Charcot osteoarthropathy is most overlooked serious limb-threatening complication of diabetes - Prof.Bilal Bin Younis**

**New Horizons in Management of Type 2 Diabetes**

**Dr. Hamed Farooqi** from UAE spoke on new horizons in the management of Type-2 diabetes. He opined that we need to achieve better blood glucose control to avoid the cardiovascular complications. Early intensive therapy for strict glucose control lowers diabetes related complications. It is important to look and prevent the major risk factors. Blood glucose control will help us. UKPDS showed that with good blood glucose control there was 43% reduction in peripheral vascular disease, 37% reduction in micro vascular disease. At present we are far from perfect blood glucose control and 40-70% of diabetics are not meeting the HbA1c targets. Almost 85% of these diabetics are overweight which is known as diabesity, 71% have high cholesterol. Type 2 diabetes has two fold greater risk of Cardiovascualr disease.

**Do not use SGLT2 drugs in those patients who are taking loop diuretics and have heart failure-Dr. Hamed Farooqi**

Speaking about the characteristics of an oral anti hyperglycemic agent, Dr. Hamed Farooqi said that it should be effective in lowering HbA1c, no hypogylcaemic episodes, no weight gain and it must reduce Cardiovascualr risk. They must be effective in people with lipids and high blood pressure. There should be few or no side effects and they must be safe. When Metformin alone is not controlling the patients, add another drug. If after three months there is no control opt for dual therapy. Assess if the patient has cardiovascular disease, the drug chosen should reduce it as well. Wait for three months and if no control, add yet another drug. Dapagliflozin inhibits SGLT2 by an insulin independent mechanism to remove excess glucose in the urine. SGLT2 inhibition requires sufficient kidney function. Its efficacy is reduced if the patient has moderate renal impairment. It is not recommended for use in patients with moderate to severe renal impairment. Patients treated with Dapagliflozin have shown stable eGFR for two years. There is major focus on dual therapy since the patient is not ready for injectable. It is difficult to convince the patients. All new drugs Dr. Hamed Farooqi said should have cardio protective and Reno protective effects. Weight loss is sustained for more than two years with GLP-1 RAs and SBP is also reduced. In those patients who are already taking CVD drugs, there is little reduction in blood pressure and there is no need to reduce the dosage of blood pressure drugs. There is no risk of hypotension.

SGLT2 lower HbA1c and also offer other benefits like weight loss and blood pressure reduction. It offers a safety profile as a class but make sure that the patient has good renal function. Do not use this drug in those patients who are taking loop diuretics and have heart failure. Euglycemic DKA has also been experienced and it can happen. UTIs and genital infections are very rare but one should be aware of it. These agents have no change in bone fractures, no involvement in amputation and no severe signal of malignancy. They do bring down the blood glucose levels. They reduce weight and there is decrease in insulin resistance as well. **Prof. Jamil Ahmad** who was chairing the session in his concluding remarks said that during the last two decades there has been emphasis on glucocentric and diabetes mellitus is seen as a complication of obesity.

**Diabetic Foot infections**

**Dr. Zulfikar G.Abbas** Chairman of Pan Asia Diabetic Foot Study Group made a presentation on Diabetic Foot Infections- Common enemy in the two continents of Asia and Africa. This session was chaired by **Prof .A. Basit** along with **Dr. Uzma Khan** while Dr**. Musarrat Riaz** was the moderator. He was of the view that identifying patient at risk of ulceration is the most important aspect for amputation prevention. Diabetic foot infection management requires a multidisciplinary team and it consists of metabolic control, relief of pressure, adequate vascular supply, empirical antimicrobial treatment, debridement, surgery and meticulous wound care.

Continuing Dr. Zulfikar Abbas said that 85% of diabetes- related lower extremity amputations are preceded by foot ulcers. Diabetes increase the risk of acquiring several serious infections and foot infections are the most common and associated with significant morbidity and mortality. Lower Limb Amputation (LLA) remains one of the most dreaded complications of diabetes. In some cultures, loss of limbs is almost equivalent of life. Approximately 40-60% of all non-traumatic amputations on the lower limb are because of diabetic foot and infections are the immediate cause of amputation in most of the patients with diabetes. Diabetic foot is equivalent to myocardial infarction and needs emergency treatment. About 15% of patients admitted in low and middle-income countries are with foot ulcers among diabetics, 33% of these patients undergo amputation while 27% of patients with foot ulcers die. Time, Dr.Zulfikar Abbas opined is tissue in the diabetic foot. There are delays in presentations to the healthcare facility and the reasons are cultural, traditional, customs, fear of losing limb, lack of awareness which is made worse due to peripheral neuropathy, lack of knowledge among health workers and there is need for early expert referral. What happens that first the patient initiates treatment at home using herbal baths and use of razor blade, then visits herbal faith healers, primary health centers, district and regional health centers and by the time the patient is finally referred to the hospital it is most often too late to save the foot or prevent death which becomes inevitable.

**Figure:**
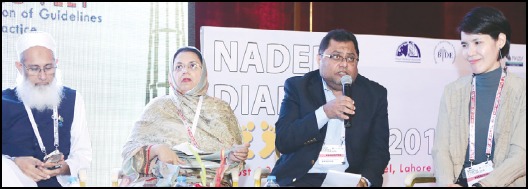
*Dr. Zulfiqar G.Abbas alongwith Prof. Basit and Dr. Gulapar chairing one of the sessions during the NADEP Foot Congress 2018*.

**Identifying patient at risk of ulceration is most important aspect for amputation prevention- Dr. Zulfikar Abbas**

Foot infections Dr. Zulfikar Abbas stated are common, complex and serious problems in patients with diabetes. In developing countries foot lesions often have underlying peripheral neuropathy and peripheral arterial disease. Infections results in prolonged hospital admission which increases medical costs. Diabetic foot ulcers in Asia and Africa are invariably infected. Patients most often come with limb-threatening infections hence the challenge we face is to find a solution to the problems posed by diabetic foot diseases in poorly resourced countries. Speaking about the natural history of diabetic foot he mentioned high-risk foot which leads to ulcerated foot, then infected foot which is followed by necrotic foot. He then showed a number of diabetic foot infections and discussed what to do in such cases. Should we culture the wound, how to get the specimen, what are the likely pathogens, does it need any surgical procedures and how to diagnose the cases of osteomyelitis? Infection, he further stated, is the main pathway to necrosis. In such patients, the host response is reduced hence early diagnosis and intervention become necessary. Infections usually begin with a break in the skin followed by colonizing of the skin flora. The predisposing factors include peripheral neuropathy, peripheral arterial disease, immunologic, metabolic and biomechanics. Peripheral neuropathy remains the major risk factor for diabetic foot ulcers. Diabetics have significant peripheral neuropathy, plantar, medial and lateral surfaces of the foot. The architecture of the foot is deformed due to an imbalance of intrinsic muscles. Diabetic neuropathy results in alterations in microvascular blood flow, the foot become warm, dry and results in the breakdown of the skin. There is a loss of protective sensation and low trans-cutaneous oxygen tension is significantly independent risk factors for foot ulcers. Diabetes limits blood collateral circulation and all this inhibits healing of infections while foot deformity increases the ulcers.

**Diabetic foot infection management consists of metabolic control, relief of pressure, adequate vascular supply, antimicrobial treatment, debridement, surgery and meticulous wound care**

Speaking about the diagnosis Dr.Zulfikar Abbas said that one should look at the lesion and see if there is any drainage, enumerate various signs of inflammation, define infection and determine the probable cause, examine soft tissues, measure the wound, evaluate neurologic status, use a probe to see if the bone can be reached. Do culture and have plain X-ray which will also provide a lot of useful information. The absence of pain, erythema, and the fever has reduced the inflammatory response. Obtain good history and ask the patient for fevers, chills and night sweats. Examine the patient, limb, and wound. Look if the tissue is involved, look for local signs i.e. redness, indurations, tenderness warmth, systemic infections and metabolic derangement. Most important clinical categories include cellulitis and deep soft tissue infections besides chronic osteomyelitis in which bones are also involved. Deep soft tissue infections involve deeper structures, necrotizing may be life-threatening whereas debridement could be life-saving, he remarked.

Management of diabetic foot infections Dr. Zulfikar Abbas said requires a multidisciplinary approach. Short term tight control helps in wound healing. This is achieved when normal blood glucose control has been achieved and optimal nutritional status is desirable to improve wound healing. Pressure shoes should be avoided. Antibiotics alone, he cautioned, will not help, one has to liaison with the surgeons and culture is important for good management. He then referred to safety and cost-effectiveness, management of topical superficial infections, oral well supplemented medical management of infections, and use of intravenous parenteral injections. Some patients may need these antibiotics for one to two weeks but in some one might have to continue these antibiotics for four weeks. All infected necrotic base in the foot must be removed. If no surgery, then antibiotics might have to be continued for three months.

He concluded his presentation by stating that to prevent foot problems, foot examinations should be performed at least once a year in patients with diabetes and more frequently in those at high risk of foot ulceration. Education is an integral part of prevention; it should be simple and repetitive. Education should be targeted at both healthcare providers, patients, and their families. Efforts are needed to increase awareness of diabetes and its complications among healthcare workers as well as patients. Early detection and treatment of diabetes will improve the course of the disease and reduce morbidity and mortality. He laid emphasis that one should examine the patient as a whole rather than looking for a hole in the patient.

**Figure:**
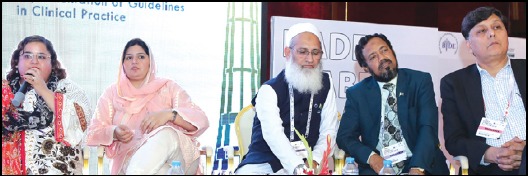
*Prof. Jamil Ahmad alongwith Prof. Basit and Prof. Qazi Masroor chairing of the sessions on Injection Techniques during the NADEP Foot Congress 2018 while photographed on right are the two speakers in this session, Ms. Erum & Dr. Musarrat Riaz*.

**Injection Techniques for Insulin**

Forum for Injection Techniques (FIT) Pakistan formally launched its Guidelines on Injection techniques during the Congress. FIT, it may be mentioned here is an initiative by BD one of the pharmaceutical company working in the field of Diabetes Care since 1924. Salient features of these guidelines were shared with the participants by **Dr.Musarrat Riaz** Consultant Endocrinologist at BIDE and Assistant Professor of Medicine at Baqai Medical University. Insulin, she pointed out, was discovered by Banting and Best in 1921. Before insulin was discovered every one with Type1 diabetes used to die within weeks or months. Animal insulin was discovered in 1922 which was followed by Human insulin in 1982 and it was followed by different analogues of insulin in 1996.

At present the prevalence of diabetes in Pakistan is about 26.3% while another 14.4% were pre diabetics. Only 3-4% of diabetics are using insulin though about 10% of diabetics should be on insulin therapy. There is no alternative to Insulin for Type-1 diabetes and Type-2 diabetics will also need insulin after few years. T2DM is progressive disease and overtime oral agents can no longer control glucose in most patients. Maintaining near normal glucose control is important to ensure maximum outcome. Speaking about the fears and concerns about insulin therapy Dr.Musarrat Riaz mentioned lifestyle factors, Lack of support, myths about insulin besides cost. Common concerns of physicians include discomfort with insulin initiation, fear of needles and it was also time consuming. FIT Pakistan Guidelines have been prepared to promote best practice in injection techniques for all involved in diabetes care. This document was prepared by a multidisciplinary team based on evidence and consensus and it has been thoroughly reviewed by leading experts of the South Asian Region. FIT Core Team consisted of Prof.Abdul Basit, Dr. Musarrat Riaz and Ms. Erum Ghafoor. The topics it covers include types of insulins available in Pakistan, insulin delivery devices i.e. syringes and pens, insulin injection techniques, insulin injection in special situations, insulin storage, insulin handling during travelling besides myths and facts about insulin injections. Insulin can be stored in refrigerator, in coolers and even in *Matka* in the villages She concluded her presentation by quoting Elliot Joslin who in 1923 said, “Everyone knows it requires brains to live long with diabetes, but to use insulin successfully requires more than brains and Insulin is a remedy primarily for the wise and not for the foolish, whether they be patients or doctors.”

**Only 4% diabetics are using insulin though 9-10% should be on insulin therapy-Dr. Musarrat Riaz**

**Ms. Erum Ghafoor** then demonstrated identification of injecting areas and injecting site assessment. Insulin can be injected in Abdomen, Thighs, Buttocks and Arms. The recommended injection site, she stated, must be inspected before each injection, site should be changed after every three to six months. Insulin is absorbed fastest from abdomen, a little slower from the arms, even more slowly from the legs and slowest from buttocks. She also discussed insulin injection in special situations like pregnancy, elderly patients, obesity besides in children and adolescents. Complications of injection techniques include lipodystrophies, hypoglycemia and bruising. While using pens, it is important to roll them few times between palms to warm insulin to get on room temperature. These guidelines in Pakistan have been published through the courtesy of PharmEvo Pharmaceuticals.

**Peripheral vascular interventions**

**Prof. Amber Malik** from Sheikh Zayed Hospital Lahore made a presentation on Recent Vascular interventions on the last day of the conference August 12^th^ 2018. This session was chaired by Prof.Basit along with Prof.Bilal Bin Younis. PAD, she said, is the disease of limbs, neck carotid, renal, aorta and diseases of veins. Almost 80% of our work at SZH, she said, is of coronary. Almost 63% of PAD patients have poly vascular disease. The causes include atherosclerosis, thrombo embolism, and vasculitis. There was a time that people used to say that there is no PAD in Pakistan but Prof.Javed Akram and colleagues in their study reported that almost 60% of diabetic patients have PAD.

**PAD has 30% mortality, 25% patients live for ten years once it is diagnosed-Ambar Malik**

She then talked about pre and post ulcer conditions. Patients sit on these ulcers, non healing ulcers. Speaking about the symptoms of PAD, she said, it is almost none. Rest at pain is critical limb ischemia. She then talked about physical findings, differential diagnosis of leg pain. Investigations include ABI, CT angio, MRA and conventional angiography. Most often we do CT. Ankle Brachial Index is also quite useful. PAD has almost 30% mortality and 25% of people live for ten years once PAD is diagnosed while 30% survive after amputation while 20% are dead. Smoking, diabetes mellitus, hypertension, fibrinogen, C reactive protein and alcohol are some of the risk factors for PAD. Management of these patients requires team work which includes vascular surgeon, orthopaedic surgeon, Diabetologist, physician, radiologist, Nephrologist and podiatrist. At times there is a perception that bleeding patients can die on the table. We use balloons and stenting to manage these patients for which donors help us a lot since patients cannot afford this expensive treatment.

Continuing Prof. Ambar Malik said that while examining patients always ask even the women patients do you smoke? Below knee disease fix up requires lot of hard work. Those patients who do not have this are lucky. Speaking about treatment she discussed in detail the medical treatment, risk of medications, platelet inhibitors, Statins, antibiotic therapy, smoking cessation, good glycaemic control. She then referred to treatment options in acute limb ischemia. Now PAD has increased and more than 50% of patients with diabetes have this disease. Patients come with atherosclerotic complications and we have to look for it to see it. With the increase in life expectancy, we see these diseses much more but intervention is only for acute conditions. Many patients will do well on medical therapy and standard exercise therapy. She then showed the management of quite a few very complicated cases with excellent results.

Participating in the discussion **Dr.Zahid Miyan** remarked that we can do lot of work together. We lack behind in vascular interventions. We all need to work as a team. Prof. Ambar Malik remarked that management of this patient is not a paying proposition. Funding is extremely important. Many things needed are not available to us at Shaikh Zayed Hospital Lahore and it is not feasible as well. Marketing of these devices is not economical hence the companies do not market it. **Prof.Basit** said that we see lot of PAD since more people with diabetes now live longer. We have started looking for it. We need to take up this challenge of PAD in diabetics for which we need to work together. It was also pointed out that use these interventions only if the coronaries are good enough otherwise you will kill the patients with MI. **Dr. Uzma Khan** remarked that almost 95% of PAD in USA are diabetics. There is a diabetic clinic at SZH Lahore and we can start a PAD registry. **Prof. Bilal Younis** said that they can help start a Diabetic Foot clinic at SZH. We should work on PAD and Diabetic Foot together.

**Ms. Barbara Eichorst** from USA through video link highlighted the importance of nutrition in the healing of diabetic foot ulcers. Speaking about risks associated with wound healing she mentioned altered immune functions, malnutrition and poor glycaemic control. Increasing micro and macro nutrition, achieving and maintaining healthy body weight, good glycaemic control, nutrition dense food, assessing treating and monitoring medical nutrition therapy can maximize wound healing. On the other hand compromised immune function affects wound healing. Nutrient deficiencies, she further stated, can affect the complex process of wound healing in multitude of ways. She also talked about how to maximize diabetic foot healing by meeting the nutritional needs of these patients’ i.e. ensuring adequate protein, calories, vitamins, minerals which all help maximize immune function. The patients need 25-35 calories per KGBW. They also need enough vitamins and minerals A, B, Magnesium, Copper and Zinc micronutrients. If the patients are not getting it through their diet, one should use these supplements. High blood glucose will lead to diabetic foot ulcer and it will be susceptible to infection. High HbA1c will also affect wound healing. Any amount of carbohydrates, she said, affects blood glucose. Medical nutrition therapy, medications, insulin will help optimize glycaemic control. She then highlighted the importance of weight management, weight reduction which also reduces calories. Hence one needs to be careful as it can also have negative effects on wound healing process. Use of Vitamin A, C, Magnesium Copper and Zinc will increase wound healing. These patients also need counseling about their eating habits. Amino acids, Omega 3 fatty acids are good for diabetic foot ulcer healing. It is important to restore nutrition intake, assess calorie intake, heal ulcer and maintain skin integrity besides supporting immune system. Nutrition is a critical concept of healing diabetic foot ulcers. She concluded her presentation by quoting Bill Gates who said, “Treatment without prevention is simply unsustainable”.

**Figure:**
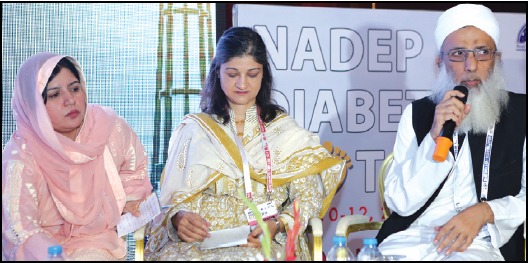
*Prof. Yakoob Ahmedani alongwith Dr. Uzma Khan & Dr.Musarrat Riaz chairing one of the sessions during NADEP Foot Congress 2018 held at Lahore*.

**Dr. Uzma Khan** from USA talked about “Neuropathic Pain- Thinking outside the Box”. She discussed in detail what we see, what we don’t and what we usually miss in chronic pain. People have diabetes and those who also suffer from neuropathy have worse quality of life. She emphasized the importance of careful history taking, duration of diabetes, drug related and toxin related pin, anti TB drugs use and use of cancer therapy. Almost 30-50% of diabetics have peripheral neuropathy. Among the metabolic conditions it is most common in diabetes. She then also mentioned about prediabetes, chronic kidney disease, chronic liver disease, use of certain medications like antipsychotics, Statins etc. Investigations include fasting blood glucose, HbA1c, also consider oral glucose tolerance test. In pre diabetics look for endocrine conditions neuropathy, hyperparathyroidism, Cushing’s syndrome, Acromegaly is rare. High level of vitamins is a problem. Lead toxicity, Dr.Uzma Khan stated is a big problem. Celiac disease and gastric bypass after ten to fifteen years can also lead to neuropathy. Check Vitamin D deficiency, arsenic in ground water. Vitamin D is effective in improving quality of life in painful neuropathy. Vitamin B 12 deficiency occurs after ten to twelve years use of Metformin. Vegetarians also suffer from Vitamin B12 deficiency and they are prone to neurogenic disorders. She also talked about patient’s concept of health, access to healthcare and cost of healthcare which were some of the challenges faced by health providers. She concluded her presentation by quoting Rumi “Why should I stay at the bottom of the well when a strong rope is in my hand”. Let us look at what is available and at hand, she added.

**Celiac disease and gastric bypass after ten to fifteen years can also lead to neuropathy - Dr. Uzma Khan**

During the discussion it was pointed out that only use those medications which have been proven effective after scientific studies. Look for other causes of neuropathic pain and for treatment only drugs should be used. Pain control center exists in the brain and pain is different in diabetics and non-diabetics. Prof. Basit remarked that neuropathy increase diabetic complications and despite best control 50-60% diabetics will have peripheral neuropathy. It is a painful condition and there are lots of medications which work. However, it is important to exclude all other causes. Improvement of Vitamin D levels help in reducing the pain but it will not eliminate the pain altogether.

